# Surveys on therapeutic effects of “halotherapy chamber with artificial salt-mine environment” on patients with certain chronic allergenic respiratory pathologies and infectious-inflammatory pathologies

**Published:** 2014

**Authors:** H Lazarescu, I Simionca, M Hoteteu, A Munteanu, I Rizea, A Iliuta, D Dumitrascu, E Dumitrescu

**Affiliations:** * National Institute of Rehabilitation, Physical Medicine and Balneoclimatology, Bucharest, Romania

**Keywords:** Halotherapy, bronchial asthma, inflammatory process, therapeutic effects

## Abstract

Halotherapy (HT), derived from speleotherapy in salt mines, is also a drug-free therapeutic method. HT effects vary depending on the therapeutic method and the structure of halotherapy environment.

The purpose of this article is to show the HT effects of “halotherapy chamber with artificial salt-mine environment” of the National Institute of Rehabilitation, Physical Medicine and Balneoclimatology (INRMFB), on patients with bronchial asthma and other chronic, infectious-inflammatory and allergic respiratory diseases, describing the clinical effects on certain nonspecific resistance factors, on markers of inflammatory processes and on certain immunological changes.

Patients were clinically assessed, with the application of hematologic investigations, analysis of nonspecific resistance to infection and of inflammatory process markers, immunologic assessments, analysis of sodium and potassium concentrations, of mineralocorticoid function and other biochemical tests.

For the experimental HT therapy performed in the “halotherapy chamber with artificial salt-mine environment” of INRMFB, 15 patients suffering from bronchial asthma, allergic rhinitis, chronic bronchitis, chronic obstructive bronchopneumopathy were selected, based on specific medical indications and contraindications and applying ethical principles, as well as 4 patients with similar pathologies for the control group, who underwent in-home drug treatment.

After the specific halotherapy treatment on patients with bronchial asthma, chronic bronchitis and chronic obstructive bronchopneumopathy, which also showed other chronic, infectious-inflammatory and allergic respiratory pathologies, triggering of anti-inflammatory (and also anti allergic) mechanisms and healing effects on inflammatory process were noted. Data acquired also proved the halo therapeutic effect causing the reduction of sensitiveness of body in patients with bronchial asthma.

**Abbreviations:** HT=Halotherapy, INRMFB=National Institute of Rehabilitation, Physical Medicine and Balneoclimatology

## Introduction

Halotherapy, derived from speleotherapy in salt mines, is also a drug-free therapeutic method, applied especially on patients with bronchial asthma and chronic bronchitis. Following the “survey for the innovative use of potentially therapeutic salt-mine environment factors, in health and balneoclimateric tourism; modeling solutions”, the conceptual model was elaborated, subsequently converted into an experimental functional model entitled “halotherapy chamber with artificial salt-mine environment”, destined for surface halotherapy and built within the National Institute of Rehabilitation, Physical Medicine and Balneoclimatology. 

This model was followed by surveys in the underground salt-mine environment, destined to assess the presence and quality of factors with speleotherapeutic / halotherapeutic potential, medical-biological multi-discipline surveys, including organismic and cellular-level analysis, before and after the experimental halotherapy treatment, on lab animals – Wistar rats with pathology experimentally induced by sensitization with ovalbumin.

Based on the experimental results acquired [**1**,**2**], the “Inception medical indications for the selection of patients with certain chronic respiratory pathologies for experimental halotherapy treatment” were elaborated.

Notably, in the infectious–inflammatory or allergic process, various systems and mechanisms of the body and organismic or cellular components were involved.

The phagocytosis process is one of the promptest defensive mechanisms against infection. The phagocytic cells are generated from precursor cells of bone marrow and they divide into macrophagous and microphagous cells. In blood, macrophagous cells are represented by monocytes, and microphagous cells – by polymorphonuclear neutrophils (PMN), which account for app. 60% of leukocytes and which are also the most significant phagocytic cells [**4**].

## Material and Methods

The selection of patients intended for the application of experimental HT treatment in the “halotherapy chamber with artificial salt-mine environment” of INRMFB was made based on medical indications and contraindications obtained, including: 

**I. Indications**

1. Patient age: 7-60 years old; infants under 10 years must be accompanied by parents. 

2. Gender: female or male.

3. Non-allergic / intrinsic / non-atopic asthma, allergic / extrinsic / atopic asthma, asthmatic bronchitis:

(1) With negative allergological tests or atopic skin tests (for non-allergic / intrinsic / non-atopic asthma) or positive tests (allergic / extrinsic / atopic asthma);

(2) eosinophils (Eo) – high or normal count in blood and ∕ or in expectorated sputum;

(3) relatively non-severe clinical evolution;

(4) asymptomatic period with or without bronchial obstruction, without asthmatic seizures; 

(5) symptomatic period with proven bronchial obstruction, without asthmatic seizures or severe asthmatic conditions; 

4. intermittent bronchial asthma, without medication or with symptomatic medication (patient’s medication):

(1) with no asthmatic seizures or severe asthmatic conditions; 

(2) with no negative evolution (frequent seizures or severe asthmatic conditions) caused by physical stress (Exercise-Induced Asthma);

(3) with no negative evolution (frequent seizures or severe asthmatic conditions) caused by humidity, changes in air temperature, gases of other microclimatic parameters (Airways Asthma);

(4) with or without non-allergic rhinitis;

(5) with or without nasal polyposis; 

(6) with or without sensitiveness to aspirin or other drugs;

5. chronic bronchitis, asymptomatic or symptomatic period without a negative evolution, without medication or with symptomatic medication (patient’s medication);

6. chronic obstructive bronchopneumopathy (BPOC), asymptomatic or symptomatic period without a negative evolution (no aggravation / acute exacerbation, no wheezing respiration, persistent cough with the production of sputum and shortness of breath), without medication or with symptomatic medication (patient’s medication);

7. allergic and chronic, infectious-inflammatory rhinitis and sinusitis, intermittent or slightly persistent, asymptomatic or symptomatic period without a negative evolution, without medication or with symptomatic medication (patient’s medication);

**II. Contraindications:**

1. Complications of asthmatic seizures (status asthmaticus, atelectasis - relatively frequent, mediastinal and subcutaneous emphysema, cor pulmonale, pneumothorax, respiratory acidosis).

2. Severe persistent form of bronchial asthma, with no drug control.

3. Asthma with continuous dyspnea and severe asthmatic conditions with violent, subintrant seizures, with a duration of 12-48 hours, treatment-resistant, no cough and expectoration, with: polypnea, asphyxia, cyanosis, vascular collapse, drowsiness. 

4. Bronchial asthma with negative evolution (frequent seizures or severe asthmatic conditions) caused by physical stress (Exercise-Induced Asthma). 

5. Bronchial asthma negative evolution (frequent seizures or severe asthmatic conditions) caused by humidity, changes in air temperature, gases of other microclimatic parameters (Airways Asthma).

6. Severe medication-related complications (severe bronchospasm, asphyxia, severe allergic reaction, anaphylactic reaction and swelling Kwinke, anaphylactic shock, status asthmaticus).

7. Acute bronchitis. 

8. Heart failure II – III.

9. Tuberculosis. 

10. Sub-compensated and decompensated cardiopathies. 

11. Cardiosclerosis. 

12. Hypertonia II-III.

13. Acute renal diseases, lithiasis, enuresis. 

14. Acute hyperacid gastritis.

15. Hepatitis or acute cholecystitis. 

16. Diabetes, severe form.

17. Colagenosis, acute rheumatic diseases. 

18. Cerebral trauma, neuroinfections, cerebral dysfunctions / central or peripheral neurologic diseases, epilepsy. 

19. Otitis, acute internal ear diseases.

20. Claustrophobia, depressions, neurosis. 

21. Emphysema and BPOC complications (acute respiratory failure with acute infections, chronic cor pulmonale / right side ventricular hypertrophy due to pulmonary hypertension, pneumothorax).

22. Post-surgery period up to 2 months. 

23. General contraindications for referral to balneary treatment and physiotherapy.

**Fig. 1:**
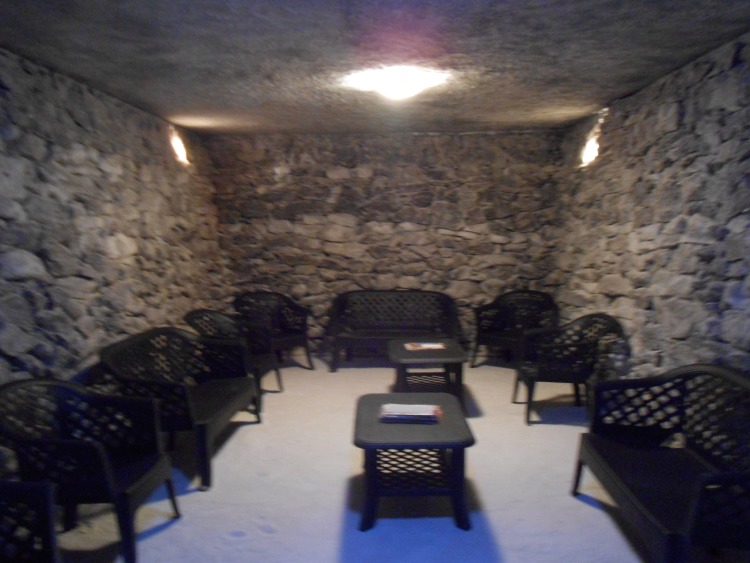
Halotherapy chamber with artificial salt-mine environment” of INRMFB, Bucharest, 11A, Ion Mihalache Blvd.

When patients for the experimental HT treatment were selected, 18 patients were investigated (16 adults and 2 infants with ages between 6–13 years) with bronchial asthma and chronic bronchitis, chronic obstructive bronchopneumopathy, of whom 14 had bronchial asthma with therapeutic control and partial control (atopic - 2, mixed- 12; moderate - 2), also suffering from other pathologies like allergic rhinitis (including moderate persistence), rhinosinusitis, respiratory virosis, viral post-pneumonia condition, chronic gastritis, duodenal ulcer, ischemic coronary disease, light mitral failure, atopic dermatitis, chronic bronchitis, hypertensive cardiomyopathy, HTAE 1-2 degree, lumbar discopathy, cervical spondylosis, osteoporosis, hypothyreosis, hemorrhoid disease, dyslipidemia, obesity III degree, urinary infection; 4 patients with chronic bronchitis or chronic obstructive bronchopneumopathy (II and III) with acute exacerbation of GOLD (1 case), showing arrhythmia, tachycardia, dyslipidemia. 

Subsequently, all the patients underwent medical-biological investigations. 

After medical and medical-biological investigations, 15 patients (suffering from bronchial asthma, but also from allergic rhinitis, chronic bronchitis, chronic obstructive bronchopneumopathy) were chosen for the experimental halotherapy (HT) treatment in the “halotherapy chamber with artificial salt-mine environment” of INRMFB (**Fig. 1**). 

A control group was also investigated, which included 4 patients with bronchial asthma, chronic bronchitis, chronic obstructive bronchopneumopathy, which was subject to in-home drug therapy, with no speleotherapy in salt mines or halotherapy [**3**]. 

## Results

Thus, during the first 3-7 days of HT procedure, occurrence and onset of irritative cough was noticed in 7 patients (5 patients with bronchial asthma, 1 – with chronic bronchitis and 1 – with chronic obstructive bronchopneumopathy) out of the 15 patients from the group subjected to HT treatment. The clinical adaptation of patients to the underground environment condition was found after 5–10 days of HT procedures, depending on the pathology and clinical progress of illness. After 10 days of HT procedures, a scarceness of cases and significant reduction in severity – until full regress – of irritative dry cough was seen in investigated patients, and after 12–15 of specific HT treatment – absence of dry cough and wheezing, and also for 2/3 of investigated patients (suffering from bronchial asthma, chronic bronchitis, chronic obstructive bronchopneumopathy) – rare cases of cough with viscous expectoration (1–3 expectorations in the last HT treatment procedures), and also cough with liquid expectoration and increase of expectorated sputum volume was noticed (in 3 patients with bronchial asthma and 1 patient with obstructive bronchopneumopathy). 

During the treatment period, no severe asthmatic conditions or additional infections occurred. ¼ of the investigated patients were characterized after 15 days of HT procedures, or 5–10 days after the HT treatment was ceased, or after gradually reducing the dose of specific medications (antihistaminic, bronchiolitis drugs, corticosteroids inhalers) to 20-30%. 

The patient with bronchial asthma and duodenal ulcer ceased the halotherapeutic treatment during the 3rd HT procedure, upon recommendation of the INRMFB physician, due to duodenal pains, and another patient with chronic bronchitis – during the 7th HT procedure, due to digestive issues occurred at home before the HT procedure, and resumed treatment after three days. The halotherapeutic treatment was also interrupted in one patient with bronchial asthma, during the 10th HT procedure, on the family doctor’s recommendation, due to acute exacerbation of cervical spondylosis pains. 

The data gathered proved the need for additional specific surveys on patients with chronic infectious-inflammatory and allergic respiratory diseases accompanied by other pathologies.

The average blood count of phagocytes – neutrophils PMN in patients with bronchial asthma and other respiratory allergies, as well as in patients with chronic bronchitis or chronic obstructive bronchopneumopathy was found to be lower compared to the lower limit of “normal (“reference”) values”, cases with lower test values being noted in 9 of 13 patients with bronchial asthma and other allergies (namely, for app. 2/3 of the respective patients group, P<0,05) and in patients with infectious-inflammatory bronchopulmonary diseases (chronic bronchitis, chronic obstructive bronchopneumopathy). The relative count of formasan-positive PMN neutrophil cells (in nitroblue tetrazolium test) was found to be high in blood for most investigated patients (12/13 – cases of bronchial asthma and other allergies P<0,01) and for patients with chronic bronchitis or chronic obstructive bronchopneumopathy. Thus, most of the investigated patients showed a deficit in the phagocytosis activity prior to the halotherapeutic treatment and also a decrease in the oxidative metabolism of phagocytes – granulocytes PMN in blood being noted. 

**Fig. 2:**
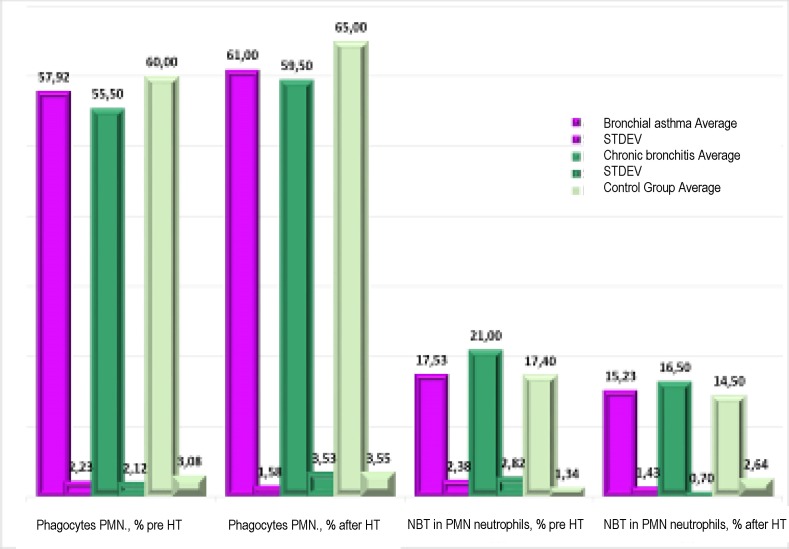
The results of the phagocytosis process for neutrophils PMN (in blood) in patients with bronchial asthma and other chronic infectious-inflammatory or allergic respiratory pathologies prior and after the experimental halotherapy treatment in “halotherapy chamber with artificial salt-mine environment” (11A Ion Mihalache Blvd., INRMFB) and in control group patients

Based on data acquired after the experimental halotherapy (HT) treatment, the triggering of non-specific resistance parameters of the body was noted (phagocytosis of neutrophils PMN, intra-cellular redox of neutrophils in NBT test) in patients (including infants) subjected to specific halotherapy treatment, compared to “normal values” and to values found in control patients, respectively with chronic respiratory pathology and drug treatment (P>0,05<0,1 and P>0,1).

The results obtained showed the positive effect of experimental halotherapeutic treatment related to the stimulation of phagocytosis process and the increase in non-specific anti-infection resistance of the body, a fact noted based on the ascendant trend of phagocytes PMN in blood and the activation of oxygen-dependent bactericide action of granulocytes PMN (nitroblue-tetrazolium test) in patients with bronchial asthma and allergic rhinitis and in those with chronic bronchitis, chronic obstructive bronchopneumopathy (P>0,1). Still, the fact that the low number of patients with chronic bronchitis and chronic obstructive bronchopneumopathy did not allow the mentioning of significant positive changes should be mentioned, and, therefore, in this case, the extension of the respective surveys is needed.

## Conclusions

The assessment of results achieved in the investigated patients with bronchial asthma, chronic bronchitis and chronic obstructive bronchopneumopathy, after a specific halotherapy treatment, indicates the triggering of an anti-inflammatory (including anti-allergic mechanisms) mechanism and a decreasing trend of the inflammatory process.

Data acquired indicate a decrease in the body’s sensitiveness and in infectious-inflammatory process in patients with bronchial asthma after HT treatment, and it also proves the need to extend the period or to repeat the halotherapeutic treatment. 
